# Haematology in the UK: A 60‐year personal perspective

**DOI:** 10.1002/jha2.213

**Published:** 2021-05-18

**Authors:** Allan Victor Hoffbrand

**Affiliations:** ^1^ Department of Haematology Royal Free Hospital University College London London UK

## Abstract

The advances in understanding the pathogenesis, in the diagnosis and classification of blood diseases and in their treatment that have been achieved over the six decades from 1960 to 2020, are reviewed. Emphasis is given to the new techniques, especially in immunology and molecular biology, that have enabled this remarkable progress. The review also highlights the major contributions of UK haematologists and non‐clinical scientists to these advances.

## 1 INTRODUCTION

The recent Special Issue of the British Journal of Haematology (BJH) provided a series of articles written by members of British Society for Haematology (BSH) to celebrate the Society's 60th Anniversary [1]. That Special Issue is supplemented here with a personal review of some of the major changes in the laboratory diagnosis, in the understanding the pathogenesis of blood and bone marrow diseases and in the improvements in their treatment that have occurred during my career which began in 1962, just 2 years after BSH, was founded. The role of British haematologists and scientists who contributed to these advances, many of whom were colleagues and close friends, will be highlighted.

As a medical student in Oxford in 1956, I attended the superb pathology course run by Howard Florey. James Gowans, a post‐doctoral research scientist working in the William Dunn School of Pathology, lectured about the lymphocyte circulation in rats which his research had unravelled. He had established that most lymphocytes were long lived, a new concept at that time. His studies had shown that lymphocytes entered lymph nodes and other lymphatic tissues from the blood, then the lymph ducts and re‐circulated into the blood stream via the thoracic duct. He then showed that lymphocytes interact with antigens and were important in antibody formation and in immunological memory. Although not taught by Macfarlane Burnet, the Nobel Prize winner based in Melbourne, I was excited to read his recently published monograph on clonal selection. The research of Gowans and Burnet was pivotal to our current knowledge of how the immune system works in combating infection. Their research also provided a scientific basis for the pathogenesis of autoimmune diseases and of clonal diseases of the lymphoid system such the lymphomas and lymphatic leukaemias as well as of the other disorders of the immune system such as immune deficiencies, graft rejection and graft versus host disease. For me it provided the stimulus in 1958 to write an essay suggesting an autoimmune basis of myasthenia gravis. This essay won the George Riddell Neurology Prize at The London Hospital and earned me £10. Simpson [2] is credited with the first publication of this theory.

**FIGURE 1:**
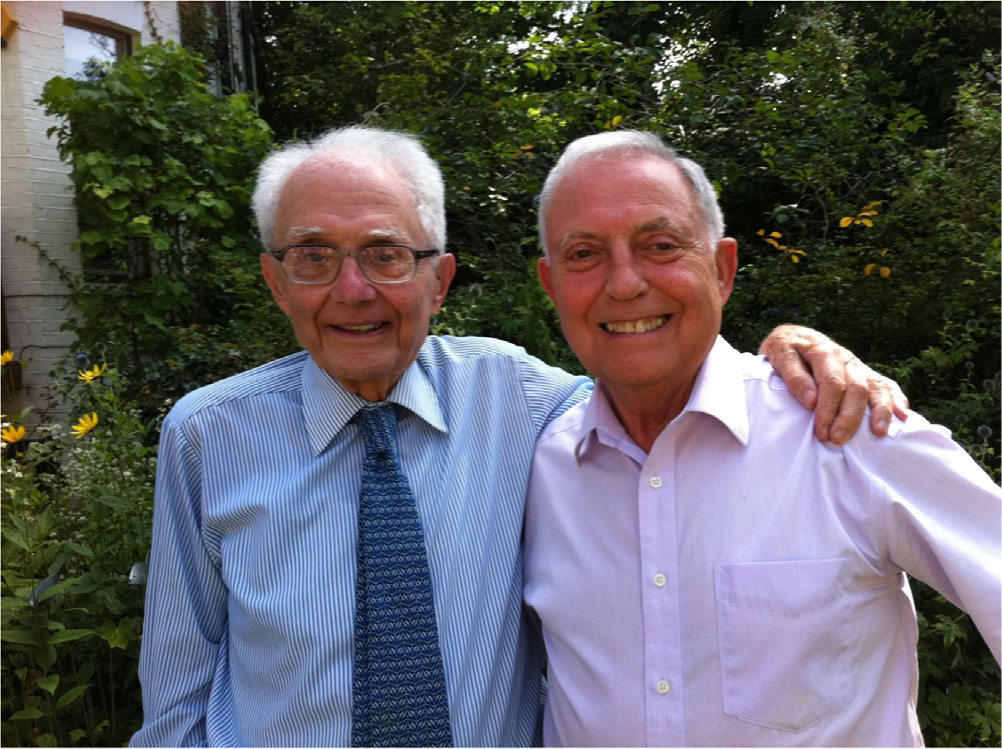
AVH with Gustav Born, who lectured to AVH at Oxford in 1956, at Born's 90th birthday party, April 2011

Gustav Born also lectured to us on Florey's course (Figure 1). He described his research on platelet function revealed by his newly described platelet aggregometer. The easy accessibility of blood which with a little test tube manipulation could provide a population of cells of one type which could be studied biochemically, immunologically and functionally, as in Born's platelet research, highlighted haematology as an attractive specialty for the clinical academic career to which I aspired. Robert Gwyn Macfarlane and Rosemary Biggs also lectured to us about the new knowledge of the coagulation cascade that they had unravelled [3]. The coagulation proteins and their role in health and disease appeared to me, however, a less exciting field than the diseases of the blood cells. Nevertheless, when I wrote a DM thesis on the red cell folate assay, I based its structure on Gwyn Macfarlane's thesis for Doctor of Medicine which in 1938 had won the Gold Medal of the University of London.

By 1957 I was a clinical medical student at the London Hospital, and here, I attended a lecture by Professor John Dacie (later Professor Sir John Dacie, FRS), on the haemolytic anaemias. Dacie described his research into the biochemical and immunological processes underlying the different haemolytic anaemias. Again I was impressed by how quantitative measurements could be easily made in a single (red) cell type from a blood sample. Also, that the course of these anaemias, and of other blood diseases, could be followed from repeat, easily obtained blood samples.

Sixty years ago advances haematology were achieved mainly by biochemical research into red cell disorders. Since then immunological, cytogenetic and more recently molecular genomic techniques have extended understanding of blood diseases, their pathogenesis, subtypes and classification, not only for the red cells but also for the inherited and acquired diseases of the white cells, platelets and of coagulation. This has led to the new treatments and supportive care that have enormously improved survival and rates of cure for almost all these diseases.

Sidney Brenner, Nobel Laureate, is quoted as saying that ‘advances in science occur by new techniques, new discoveries and new ideas probably in that order’. The advances in all areas of haematology since the early 1960s have indeed largely been driven by new techniques including automated cell counters, flow cytometry, immunohistology, monoclonal antibodies, cytogenetic and molecular tests ranging from the Giemsa banding technique, fluorescent in situ hybridisation (FISH), Southern blotting, Sanger sequencing, the polymerase chain reaction (PCR) to next generation sequencing (NGS).

The role of British haematologists and non‐clinical scientists in contributing to these advances is described next with particular emphasis on some of the topics not covered in the Special Issue.

## 2 RED CELL DISORDERS

Major contributions to the understanding of the pathogenesis of the haemolytic anaemias were made by John Dacie who first described several of the diseases such as microangiopathic haemolytic anaemia [4], performed many of the original studies which defined these diseases and who comprehensively reviewed in five books everything then known about them, with examples from the many patients he studied. Sheila Worlledge, a close colleague of Dacie, contributed substantially to knowledge of the autoimmune haemolytic anaemias and together they described the first drug‐induced autoimmune disease, autoimmune haemolytic anaemia initiated by a drug, methyldopa [5]. Pat Mollison was a major pioneer in developing blood transfusion during and after the Second World War and authored the definitive textbook on all aspects of blood transfusion. Sheila Worlledge and more recently Marcela Contreras among many others carried on Mollison's legacy of research, clinical expertise and teaching in this field.

Lucio Luzzato who succeeded Dacie at the Hammersmith Hospital in 1981 was a pioneer of research in glucose‐6‐phosphate dehydrogenase deficiency and its relation to malaria [6, 7]. He later described many of the mutations underlying the deficiency. His second major contribution, with several colleagues including Peter Hillmen in Leeds, has been into the pathogenesis and treatment of paroxysmal nocturnal haemoglobinuria (reviewed in the Special Issue by Luzzatto and Kradimitris [8]). It is necessary also to mention here Ernest Beutler a true giant in many areas of haematology including among others haemolytic diseases, G6PD deficiency, haemochromatosis, Gaucher disease and X chromosome inactivation. To my mind Ernest Beutler was the greatest of the many great haematologist I met in my career.

Research into the nature and treatment of aplastic anaemia, now recognised as a stem cell disease, was carried out by Ted Gordon‐Smith first at Hammersmith Hospital and then with Judith Marsh at St Georges Hospital where they pioneered the use of different anti‐lymphocyte globulins and of bone marrow transplantation for this disease.

UK researchers over several decades played a pivotal role in researching and identifying the genetic disorders of haemoglobin, and in establishing the treatment and prevention of these diseases, collectively the most frequent of single gene disorders worldwide. These ‘haemoglobinopathies’ were among the first of now many blood diseases where molecular genomic research has revealed the pathogenesis. Linus Pauling‘s group in USA first showed that haemoglobin S had a different electrophoretic mobility to haemoglobin A [9]. Vernon Ingram working at the Cavendish Laboratories in Cambridge under the leadership of Max Perutz later showed that sickle haemoglobin differs from haemoglobin A by a single amino acid substitution [10, 11]. Research by Perutz for which he received the Nobel Prize in 1962 (shared with John Kendrew for his research into the structure of myoglobin) revealed the three dimensional structure of haemoglobin [12]. Hermann Lehmann based at St Bartholomews Hospital in London, was responsible for describing many of the different haemoglobin variants [13]. Several groups had concluded that the thalassaemia syndromes were due to failure of synthesis of haemoglobin but it was Weatherall, Clegg and Naughton [14] who first demonstrated unequivocally that the alpha and beta thalassaemias were due to unbalanced alpha and beta globin chain synthesis.

Weatherall and the group of talented clinical scientists (John Clegg, Douglas Higgs, Bill Wood, John Old, Swee Lay Thein and Jim Wainscoat, among many others) he gathered at the Institute of Molecular Medicine he founded in Oxford subsequently made major contributions to the discovery of the molecular mutations underlying the different forms of the thalassaemia, to the description of previously unrecognised syndromes, to carrier detection, to safe and reliable antenatal diagnosis and to prevention of births with the more severe forms of thalassaemia [15]. It is on the basis of the elucidation of the molecular mechanisms underlying the switch from fetal haemoglobin to adult haemoglobin in which Bill Wood and Swee Lay Thein made substantial contributions (reviewed by [16, 17]) that some of the current gene therapy strategies have been devised for treating sickle cell anaemia and thalassemia major [18, 19].

Propper and Nathan in Boston first proposed daily subcutaneous desferrioxamine as effective iron chelation therapy for transfusional iron load, a major cause of death in thalassaema major [20, 21]. The benefit of this approach was confirmed by further studies in London [22]. It was Robert Hider at the University of Essex who developed the first orally effective iron chelators, the hydroxypyridin‐4‐ones, deferiprone being the first orally active chelator to be used clinically (reviewed by Hider and Hoffbrand [23]). John Porter who with Ernest Huehns had been involved in early research on the hydroxypyridin‐4‐ones later played a major role in establishing deferasirox as a second and now most prescribed orally iron chelator (reviewed by Porter [24]).

My own research career began in 1963 in another dominantly a red cell disorder, megaloblastic anaemia. Much had already been established in this field where Whipple, Minot, Murphy and Dorothy Hodgkin were previous Nobel Prize winners. David Mollin with whom I started research at Hammersmith Hospital had shown with Christopher Booth that the ileum is the site of cobalamin (vitamin B12) absorption. With Innes Ross he developed the first reliable serum vitamin B12 assay and with Alan Waters one of the first serum folate assays. My role with Beverley Newcombe was to develop a red cell folate assay and then to use this to investigate folate/vitamin B12 interactions and folate deficiency in various clinical groups [25]. At that time assays for vitamin B12 and folate were microbiological. These have now been replaced by automated chemiluminescence‐based competitive binding assays.

Later research from 1968 showed that vitamin B12 deficiency caused failure of folate polyglutamate synthesis due to a block in conversion of methyltetrahydrofolate (methyl THF) to THF, the form of folate needed as substrate for the enzyme adding extra glutamate moieties and so keeping folates inside the cell [26]. The DNA defect in megaloblastic anaemia was suggested to be failure of elongation of DNA to form new, fully double stranded DNA, after initiation of new DNA synthesis at multiple replicon origins along chromosomes [27]. Kanagasabai Ganeshaguru and Edith Tripp had established an assay to measure for the first time in human cells the levels in bone marrow of the immediate DNA precursors, the deoxynucleoside triphosphates [28]. Two years later we adapted this assay to measure instead of the substrates of the reaction, the activity of the enzyme terminal deoxynucleotidyl transferase (TDT) which uses these precursors to synthesise DNA. This switch of the biochemical assay enabled us in 1976 to change the focus of our research from a mainly red cell disorder megaloblastic anaemia to white cell in the fields of leukaemia and lymphoma [29]. This change of focus coincided with a move in 1974 from Hammersmith to the Royal Free Hospital.

Like other haematologists I had considered the field of folate deficiency belonged to our specialty. The next major advance in folate research, however, was not in haematology but in maternal and neonatal aspects. This was a result of the randomised MRC funded Trial led by Nicholas Wald and colleagues (Wald [30]).This showed that folic acid prior to and during pregnancy could reduce substantially the incidence of neural tube defects (NTDs) such as spina bifida in the newborn. Studies in Dublin by John Scott's group then showed that the incidence of NTDs increased the lower maternal plasma and red cell folate levels and plasma vitamin B12 levels, even when these were in the accepted (by haematologists) normal ranges [31, 32]. Over 80 countries now fortify grain or flour with folic acid, implemented in USA since 1998, with a reduction in incidence of NTDs in every country where this has been monitored. Sadly fortification has not been introduced in the UK, the country in which the discovery was first made.

## 3 COAGULATION

The immense contribution to the diagnosis, prevention and treatment of haemophilia made by UK haematologists has been summarised by Nathwani and Tuddenham [33]. Many of these advances were made in the Katherine Dormandy Haemophilia Centre at the Royal Free Hospital. Katherine Dormandy herself pioneered home treatment for haemophilia, improving substantially the quality of life of the patients. Ted Tuddenham, who with Peter Kernoff succeeded Dormandy as co‐directors of the Centre, led the team that first purified factor VIII leading to cloning the factor VIII gene which paved the way for the production of recombinant factor VIII and for antenatal diagnosis by DNA techniques. Key to this breakthrough was the production of the first monoclonal antibodies to coagulation factors made by Tuddenham's group working with Alison Goodall from George Janossy's laboratory (see below). Two decades later Amit Nathwani was appointed Director of the Centre, and with Tuddenham has been the lead researcher in gene therapy for haemophila A and B using an adeno‐associated virus vector [34]. John Pasi who trained in the Centre, has led at St Barts Hospital, the first successful trial of gene therapy for haemophilia A using a vector Nathwani and Tuddenham developed [35, 36].

Among the many other contributions in the coagulation field made by UK haematologists are those by Stuart Douglas, Arthur Bloom and Leon Poller who enhanced the understanding and treatment of bleeding and thrombotic disorders and their therapy. Duncan Thomas and Trevor Barrowcliffe were pivotal in the development of low molecular weight heparins (LMWHs) [37, 38]. Based at the National Institute for Biological Standards, not far from the Royal Free Hospital, in Hampstead, London, they demonstrated the improved specificity of LMWHs, compared with unfractionated heparin, for factor Xa compared with thrombin, and the longer half‐life in plasma of LMWHs enabling efficacy of once daily injections [39]. They also showed less interaction of LMWHs with platelets, reducing the side‐effect of bleeding. I had a special friendship with Duncan as we had both been undergraduates at the same Oxford College, Queen's and met at many Old Members dinners there through the years.

## 4 WHITE CELL DISORDERS

The greatest expansion of knowledge in haematology over the last 60 years has been in the haematological malignancies. It started in 1960 at a low pace. The advances in all aspects have resulted from the application of cell biological, immunological, cytogenetic and molecular techniques. Characterisation of the human bone marrow stem and precursor cells was pioneered in the 1960s in Australia by Bradley and Metcalfe, in Canada by McCulloch and Till and in Manchester by Dexter and Lajtha [40] who developed long‐term (Dexter) culture techniques. Together these culture techniques were used to reveal the processes of normal haemopoiesis and to characterise the biological differences between the early clonal neoplastic cells in the various myeloid malignancies and the equivalent normal marrow stem and precursor cells. They also led to the identification of the growth factors involved at different stages of haemopoiesis and of erythropoietin and G‐CSF to be used therapeutically in patients with renal failure, receiving chemotherapy or stem cell transplantation or with other causes of bone marrow failure.

In the 1960s cytochemistry developed especially by Frank Hayhoe in Cambridge and then David Swirsky and Barabara Bain in London, had been a mainstay in the differential diagnosis of the different leukaemias. The 1970s saw the invention in Cambridge of monoclonal antibodies by Kohler and Milstein [41], the wide application of immunofluorescence, flow cytometry and immunohistochemistry using these antibodies in classifying the different haematological neoplastic diseases. Mel Greaves began research with Ivan Roitt at University College Hospital. With several colleagues they brought together research of many, including Max Cooper in USA and Jacques Miller at the Chester Beatty Institute for Research, London and the Walter and Eliza Hall Institute, Melbourne, by proposing the existence of two populations of lymphocytes which they named B (bursa dependent) and T (thymus dependent) [42]. Mel Greaves later pioneered in collaboration with Roger Hardisty and Judith Chessels at Great Ormond Street Hospital, the immunological classification of childhood leukaemia [43]. The classification was used as an important component of UK national clinical trials of the treatment of childhood leukaemia, initially directed by Humphrey Kay at the Royal Marsden Hospital, London [44].

George Janossy continued his collaboration with Mel Greaves after establishing a new department of immunology at the Royal Free Hospital in 1976 (e.g., Janossy et al [45]) and, as discussed below, with Ken Bradstock developed some of the first tests for minimal residual disease in acute lymphoblastic leukaemia (ALL). Immunohistology was also developed in his department as well as by David Mason, Kevin Gatter and Wendy Erber in Oxford, Peter Isaacson at University College Hospital, London, Harald Stein in Germany [46], Brunangelo Falini in Italy and by many experts including Elaine Jaffe and Nancy Harris in USA [47]. Isaacson used these and molecular techniques to reveal later the pathogenesis of gastric malt lymphoma [48].

As recently as 1970 almost nothing was known of the genetic mutations underlying the congenital and acquired diseases of the blood and bone marrow. Some chromosome abnormalities such as the Philadelphia chromosome in CML and in some cases of ALL had been described. The development in the 1970s of Giemsa banding technique, with which Janet Rowley in Chicago revealed translocations underlying chronic myeloid leukaemia [49] and translocations in acute myeloid leukaemia such as t(15;17) in promyelocytic leukaemia, led to widespread use of cytogenetic analysis for haematological diagnosis and research. In the UK Lorna Secker‐Walker, Helen Walker, Rosemary Gale, Christine Harrison, Anthony Moorman and many others made substantial contributions both in original studies and in supporting the MRC Leukaemia Clinical Trials [50]. Cytogenetics using Giemsa banding, complemented the morphological and immunological findings in the so called M (Morphological), I (Immunological) and C (Cytogenetic) classification of acute myeloid leukaemia [51]. Southern blotting introduced in 1975 [52] was used in USA by Korsemeyer and colleagues to demonstrate clonal rearrangement of the immunoglobulin genes in B cell ALL [53, 54]. Foroni et al [55] extended these findings to other B‐cell malignancies including chronic lymphocytic leukaemia and the B‐cell lymphomas in Lucio Luzzatto's laboratory at Hammersmith Hospital.

The fluorescent in situ hybridisation technique, [56, 57], and the polymerase‐chain‐reaction technique invented by the American Nobel Prize winner Kary Mullis (reviewed by Mullis et al [58]) and next generation sequencing (NGS) in the first decade of the 21st century accelerated molecular genetic research, revealing that many different mutations may underlie apparently similar congenital and acquired diseases of the bone marrow and lymphatic tissue. Also how these disorders often progressed from oligoclonal beginnings, with linear or branching clonal evolution resulting in a minor clone at presentation becoming the dominant clone at subsequent relapse. The molecular studies have also revealed the presence of clonal populations of cells in the blood and bone marrow, increasing in incidence with age, of otherwise healthy individuals. They have enabled effective chemotherapy specifically targeted at one or other point in the cell signalling pathways. The first was imatininib which blocks at the BCR‐ABL1 tyrosine kinase ATP binding site. More recently the BCL2 inhibitor Ventoclax was developed in collaboration with Andrew Roberts and others, by David Huang, who after completing his training in the UK at the Royal Free Hospital peformed this major successful research in Melbourne [59].

When I qualified in medicine at the end of 1959, all patients with acute leukaemia died of the disease, many within days or a few weeks of diagnosis. As a house physician at the London Hospital in 1960, I was told not to waste my time with a young man of 22 we admitted with acute myeloid leukaemia as there was no effective treatment, and he would be dead within a week or 2. The outlook for most of the patients with chronic myeloid leukaemia, lymphomas and myeloma was also dismal with a life expectancy of 2 or 3 years. Over 90% of patients with childhood leukaemia and Hodgkin lymphoma are now cured, and chronic myeloid leukaemia has a long‐term prognosis comparable to that of subjects without the disease. Life expectancy has also improved substantially for patients with almost all the other haematological neoplasms. Even for acute myeloid leukaemia survival has steadily improved especially in younger patients with almost 75% of those in the 0‐ to 14‐year‐old age group now surviving 10 years or more. As reviewed by Craddock [60], MRC Clinical Trials, many led by Alan Burnett, have contributed substantially to this success. Even for the elderly new regimens are at last beginning to improve prognosis [61]. Bone marrow and peripheral blood stem cell transplantation, whether allogeneic or autologous (reviewed by Barrett and Craddock [62]), CAR‐T cell and bi‐specific antibody therapy are achieving remissions in patients with relapsed and otherwise resistant ALL, NHL or myeloma.

The major role of UK haematologists in laboratory and clinical research and in clinical trials in the haematological malignancies, many of the trials under the auspices of the Medical Research Council, has been largely covered in the Special Issue. I end this review by selecting three important areas of the haematological neoplasms, not specifically included in the Special Issue, where UK research appears to me to have made a major impact.

## 5 THE PATHOGENESIS OF CHILDHOOD ACUTE LYMPHOBLASTIC LEUKAEMIA

After his earlier research on the characterisation of leukaemic cells by immunological markers mentioned earlier, Mel Greaves focussed on the pathogenesis of childhood acute lymphoblastic leukaemia (ALL), the most frequent paediatric cancer in developed countries [63]. He performed elegant informative studies of monozygotic twin pairs who developed the disease. This highly original research revealed two discrete steps in the evolution of childhood leukaemia. In the first, usually if not always in utero, a clone is formed bearing an abnormal fusion gene such as *ETV6‐RUNX1* or a clone with hyperdiploidy. This clone remains covert until after birth. Greaves and his colleagues then examined the DNA derived from blood spots on Guthrie cards and DNA from cord blood samples. These showed that abnormal clones of the leukaemia were already present at birth in the children who later went on to develop overt B‐ALL. They also showed the ETV6‐RUNX1 translocation to be present in as many as 1% of newborns, but only 1% of those positive for the chromosome translocation at birth later develop leukaemia.

The second genetic event transforms the pre‐existing clone to overt leukaemia. The event as described by Greaves [63] is ‘driven by the V(D)J‐recombination‐activating protein (RAG) and activation–induced cytidine deaminase(AID)‐driven copy number alterations in the case of ETV6‐RUNX1 translocation’. The most likely cause of this second event appears to be an abnormal immune response to infection. No specific infection has been implicated but Greaves proposed the ‘delayed infection’ hypothesis. Under‐exposure of the immune system to microbial infection in the neonate and infancy is postulated to lead to susceptibility to dysregulated responses in childhood and so trigger B‐ALL. This would explain the increased incidence of the disease in developed compared with underdeveloped societies. Also consistent with the hypothesis, first born children are more at risk than their younger siblings; those attending day care earlier in life are relatively protected. Greaves also summarises evidence derived from gene wide association studies (GWAS) for a contribution of inherited alleles in a range of genes. Acquired mutation of this same set of genes has been implicated in the pathogenesis of the disease.

Greaves has reviewed how unravelling the genetic and cellular events leading to leukaemia and lymphomas has contributed to the understanding of the complex biological events that underlie cancer more widely [64]. His outstanding contribution to knowledge of childhood leukaemia and of cancer more generally has been recognised among many other awards by the Royal Medal in 2017 and a knighthood in 2018.

## 6 MINIMAL RESIDUAL DISEASE (MRD)

The definition of remission in acute leukaemia during the 1960s, 1970s and 1980s depended on finding fewer than 5% leukaemic blasts in the bone marrow. However, even 1% leukaemic blasts could imply a total body burden of 10 × 10–10^11^ leukaemic cells. Enrichment of the cell fraction containing the blast cells and chromosome analysis using Giemsa banding or FISH could improve sensitivity of detection of the remaining leukaemic cells but not to significantly below 1%.

The first substantially more sensitive techniques were immunological. These depended on finding small numbers of cells with leukaemia‐associated phenotypes (LAPs) in the bone marrow or at other sites in ALL. TDT was the first useful immunological marker for MRD since TDT positive cells in CSF or testis; however few, in patients with ALL showed the presence of leukaemia. Moreover any cells positive both for T cell markers and for TDT in the marrow in cases of T‐ALL in remission by conventional microscopy, indicated residual leukaemia [65, 66].

Flow cytometry with double or triple colour markers, developed by Dario Campana and Elaine Coustan‐Smith first with George Janossy at the Royal Free Hospital and then at St Jude's Hospital, Memphis, is now widely used to detect MRD not only in the acute leukaemias but also in chronic lymphocytic leukaemia, lymphomas and myeloma [67]. Its maximum sensitivity is 1 × 10^4^. It has the advantage of rapid availability of the results, being widely applicable in both myeloid and lymphoid neoplasias, being cost effective and easy to perform. Change of phenotype between presentation and relapse is a potential problem but in practice this has not been a frequent cause of false negative results.

Molecular PCR‐based techniques have increased the sensitivity of detection of MRD in peripheral blood or bone marrow to 1 × 10^6^ cells. These PCR techniques were initially applied to detect immunoglobulin (Ig) and T‐cell receptor gene rearrangements in B‐cell and T‐cell ALL. The fingerprint‐like sequences were detected in presentation samples, and these sequences sought in remission blood or bone marrow [68, 69, 70]. Early studies revealed that many cases of ALL showed oligoclonality at presentation with relapse originating from one or other sub‐clone. They showed that the relapse clone was present at diagnosis. This was demonstrated using DNA obtained from scraping archived slides of presentation bone marrow which was compared to DNA of bone marrow at relapse, even 10 or more years later. Letitzia Foroni at the Royal Free Hospital focused on adult ALL [71, 72] and demonstrated that bone marrow analysis for MRD as early as day 28 or 56 after starting chemotherapy could predict future outcome in the majority of adults with ALL.

In the early 1990s Nick Cross working with John Goldman at Hammersmith Hospital had developed sensitive PCR techniques for detecting MRD in chronic myeloid leukaemia [73]. After returning to Hammersmith Hospital, Foroni performed research in CML which demonstrated that MRD analysis as early as 3 months could predict overall outcome, especially excellent outcome if patients achieved by then residual disease below 10% [74]. The Hammersmith group's research also involved the other myeloid leukaemias and the myeloproliferative diseases employing NGS to detect molecular mutations [75].

MRD monitoring, often using NGS, has now been incorporated as standard practice in treating childhood and adult ALL and AML, in CML, and in many cases of chronic lymphocytic leukaemia and myeloma. It is an essential laboratory component of virtually all clinical trials of new therapies and protocols in these diseases.

## 7 MYELOPROLIFERATIVE NEOPLASMS (MPN)

In 2005 four groups including that of Tony Green in Cambridge described the *JAK2 V617F* mutation in most patients with polycythaemia vera and about half those with essential thrombocythaemia (ET) and myelofibrosis (MF) (reviewed by Campbell and Green [76]). My claim to be associated in any way with this research is tenuous ‐ Tony had spent part of his training in haematology as a lecturer at the Royal Free Hospital. In 2013, the Cambridge group went on to describe a different class of *JAK2* mutations (in exon12) in patients who often present with erythrocytosis [77]. A minority of ET and MF (about 5%–8%) patients have a mutation of the thrombopoietin receptor gene (*c‐MPL*). In most cases lacking a JAK2 mutation, the Green team together with the Kralovics group in Vienna,[78] identified multiple gain–of‐function mutations in calreticulin (CALR) thus providing an unexpected link between endoplasmic reticulum and malignancy [79]. Mutations in these three genes (*JAK2, CALR* and *MPL*) underlie nearly all the three myeloproliferative diseases, and the presence of one or other in ET and MF has prognostic significance. These discoveries have led to the introduction of simple molecular tests that distinguish the MPNs from reactive conditions and catalysed the development of new drugs including JAK2 inhibitors such as ruxolitinib effective in these and in other diseases.

The Green group pioneered the use of clonally‐derived haemopoietic colonies to explore MPN pathogenesis and to reveal the cellular and molecular consequences of JAK2 and CALR mutations. Collaborative studies with Peter Campbell revealed additional somatic mutations which are of prognostic significance and have resulted in a new genetic classification of the MPNs as well as giving patients a personalised prognosis [80]. They and others have also shown that the MPNs are clonally heterogeneous with sub‐clones showing additional mutations in genes such as *TET2* or *DNMT3A*. The order in which the mutations are acquired was shown to determine sub‐clone size, disease presentation and progression, the first time this has been demonstrated for any cancer [81]. Most recently the Cambridge groups (now including that of Jyoti Nangalia) have shown that the JAK2 mutation may be acquired as early in life as the first or second decade but then can lay dormant until presenting with overt disease several decades later [82].

The UK Primary Thrombocythaemia 1 suite of clinical trials studying ET, were led initially by Tom Pearson and Tony Green and were opened in 1997. Following Tom Pearson's retirement, Claire Harrison and Peter Campbell joined Tony as co‐chief investigators. The High Risk trial showed that hydroxycarbamide (hydroxyurea) with low dose aspirin was superior to anagrelide with low dose aspirin for treatment of high risk ET [83]. The Intermediate Risk trial ran for 20 years and demonstrated that intermediate risk patients should not receive cytoreductive therapy [84]. Among many other clinical studies, the UK's network of MPN clinicians showed that ruxolitinib was not superior to best alternative therapy for hyroxyurea resistant ET [85]. With Donal McLornan Claire Harrison recently reviewed the use of novel agents in the MPNs [86] and with Susan Robinson, the management of the MPNs in pregnancy [87].

## 8 CONCLUSION

The last six decades have seen enormous changes in the diagnosis and management of blood diseases. These advances have been driven by new techniques, particularly immunological, cytogenetic and molecular. Diseases recognised in 1960 have been re‐classified into subtypes and prognostic groups, usually on the basis on their underlying genetic mutations. New diseases have been identified especially in the field of the haematological malignancies. Molecular techniques have also revealed the existence in otherwise healthy individuals, of clonal populations of haemopoietic cells which may be present for many years, even from childhood which may or may not progress to overt clinical disease. Haematology has been at the forefront of cancer research, exemplified by our detailed knowledge of the pathogenesis of childhood leukaemia. In this review, I have attempted to highlight the contributions of UK haematologists, many of whom have been my close colleagues and friends, to some of these advances, especially in those diseases not specifically covered in the recent 2020 Special Issue of BJH.

Chemotherapy and stem cell transplantation have dominated treatment of the blood cancers over the last six decades. The next decades promise even more improvements in cure rates and with reduced treatment‐related toxicity, especially by the use of more sophisticated antibody or cellular immunotherapy and by the more widespread use of gene therapy for treatment of both benign and malignant haematological diseases.
